# Supplementary material for: Comparison of machine-learning and logistic regression models for prediction of 30-day unplanned readmission in electronic health records: A development and validation study
Source: PLOS Digit Health. 2024 Aug 20;3(8):e0000578. doi: 10.1371/journal.pdig.0000578 (PMC11335098; doi:10.1371/journal.pdig.0000578)
Supplement: S6 Table — (DOCX) [file pdig.0000578.s006.docx]

| **S6 Table. Hyperparameters in each model for the dataset with the largest number of variables including blood-test results (1543 variables)** | | | | | | | |
| --- | --- | --- | --- | --- | --- | --- | --- |
| Gradient-boosting decision tree | | Random forest | | Deep neural network | | Logistic regression | |
| Hyperparameter | Value | Hyperparameter | Value | Hyperparameter | Value | Hyperparameter | Value |
| col sample rate | 0.8 | number of trees | 50 | epochs | 21.7 | λ for LASSO | 0.000033 |
| col sample rate per tree | 0.8 | score tree interval | 5 | Mini batch size | 1 |  | |
| fold assignment | Modulo | fold assignment | Modulo | layer | 4 |  |  |
| number of trees | 64 | stopping_metric | logloss | layer 1 units (type) | 4650 (input) |  |  |
| max depth | 15 | stopping_tolerance | 0.0017 | Regularization | NA |  |  |
| min rows | 100 | max depth | 20 | Dropout rate | 15% |  |  |
| stopping_metric | logloss | sample_rate | 0.63 | layer 2 units (type) | 100 (ReLU) |  |  |
| stopping tolerance | 0.0017 | col_sample_rate | 1 | Regularization | NA |  |  |
| distribution | bernoulli | col_sample_rate_per_tree | 1 | Dropout rate | 10% |  |  |
| histogram type | UniformAdaptive | histogram_type | UniformAdaptive | layer 3 units (type) | 100 (ReLU) |  |  |
|  | | distribution | bernoulli | Dropout rate | 10% |  |  |
|  |  |  | | layer 4 units (type) | 2 (Softmax) |  |  |

LASSO = least absolute shrinkage and selection operator.
