# Supplementary material for: Comparison of machine-learning and logistic regression models for prediction of 30-day unplanned readmission in electronic health records: A development and validation study
Source: PLOS Digit Health. 2024 Aug 20;3(8):e0000578. doi: 10.1371/journal.pdig.0000578 (PMC11335098; doi:10.1371/journal.pdig.0000578)
Supplement: S8 Table — (DOCX) [file pdig.0000578.s008.docx]

| **S8 Table. The goodness of fit indicators of each model for validation** | | | | |
| --- | --- | --- | --- | --- |
| R^2^ Cox–Snell and R^2^ Nagelkerke (the higher, the better)  Brier score (the lower, the better) | Gradient-boosting decision tree | Random forest | Deep neural network | LR-LASSO |
| Pattern 1: 102 variables, including binary variables that ≥5% of patients had, without blood-test results | 0.036 and 0.096  0.056 | 0.038 and 0.100  0.056 | 0.028 and 0.074  0.057 | 0.032 and 0.086  0.057 |
| Pattern 2: 112 variables, including binary variables that ≥5% of patients had, with blood-test results | 0.039 and 0.102  0.056 | 0.040 and 0.107  0.056 | 0.038 and 0.102  0.056 | 0.036 and 0.095  0.056 |
| Pattern 3: 296 variables, including binary variables that ≥1% of patients had, without blood-test results | 0.041 and 0.107  0.056 | 0.043 and 0.113  0.056 | 0.038 and 0.100  0.056 | 0.038 and 0.100  0.056 |
| Pattern 4: 306 variables, including variables that ≥1% of patients had, with blood-test results | 0.042 and 0.112  0.055 | 0.044 and 0.118  0.056 | 0.044 and 0.116  0.056 | 0.040 and 0.107  0.056 |
| Pattern 5: 1533 variables, including binary variables that ≥10 patients had, without blood-test results | 0.042 and 0.110  0.055 | 0.043 and 0.113  0.056 | 0.029 and 0.076  0.057 | 0.039 and 0.103  0.056 |
| Pattern 6: 1543 variables, including binary variables that ≥10 patients had, with blood-test results | 0.043 and 0.114  0.055 | 0.045 and 0.119  0.056 | 0.034 and 0.091  0.057 | 0.042 and 0.110  0.055 |

LR-LASSO = logistic regression with the least absolute shrinkage and selection operator.
