# Supplementary material for: Comparison of machine-learning and logistic regression models for prediction of 30-day unplanned readmission in electronic health records: A development and validation study
Source: PLOS Digit Health. 2024 Aug 20;3(8):e0000578. doi: 10.1371/journal.pdig.0000578 (PMC11335098; doi:10.1371/journal.pdig.0000578)
Supplement: S9 Table — (DOCX) [file pdig.0000578.s009.docx]

| **S9 Table. The calibration indicators of each model for validation** | | | | |
| --- | --- | --- | --- | --- |
| Calibration slope (the closer to the 1, the better)  Calibration-in-the-large (the lower, the better) | Gradient-boosting decision tree | Random forest | Deep neural network | LR-LASSO |
| Pattern 1: 102 variables, including binary variables that ≥5% of patients had, without blood-test results | 0.911  0.003 | 0.983  0.006 | 0.858  0.005 | 0.959  0.005 |
| Pattern 2: 112 variables, including binary variables that ≥5% of patients had, with blood-test results | 0.909  0.002 | 0.995  0.006 | 0.864  0.008 | 0.958  0.005 |
| Pattern 3: 296 variables, including binary variables that ≥1% of patients had, without blood-test results | 0.936  0.003 | 1.141  0.004 | 0.962  0.005 | 0.962  0.005 |
| Pattern 4: 306 variables, including variables that ≥1% of patients had, with blood-test results | 0.929  0.002 | 1.166  0.004 | 0.985  0.020 | 0.963  0.004 |
| Pattern 5: 1533 variables, including binary variables that ≥10 patients had, without blood-test results | 0.959  0.003 | 1.507  0.003 | 0.949  0.003 | 0.967  0.004 |
| Pattern 6: 1543 variables, including binary variables that ≥10 patients had, with blood-test results | 0.944  0.002 | 1.497  0.003 | 0.576  0.001 | 0.967  0.004 |

LR-LASSO = logistic regression with the least absolute shrinkage and selection operator.
