# Supplementary material for: Comparison of machine-learning and logistic regression models for prediction of 30-day unplanned readmission in electronic health records: A development and validation study
Source: PLOS Digit Health. 2024 Aug 20;3(8):e0000578. doi: 10.1371/journal.pdig.0000578 (PMC11335098; doi:10.1371/journal.pdig.0000578)
Supplement: S11 Table — (DOCX) [file pdig.0000578.s011.docx]

| **S11 Table. The change in the c-statistic by excluding each of the 10 variables with high variable importance in a gradient-boosted decision tree for the data table with the largest number of variables including blood-test results (1,543 variables)** | | | |
| --- | --- | --- | --- |
| No. | Excluded variable | C-statistic | Change in c-statistic |
| n/a | None (i.e., the full model with 1543 variables) | 0.764 | n/a |
| 1 | Number of hospitalization in the past year | 0.755 | -0.009 |
| 2 | Blood test: hemoglobin | 0.754 | -0.010 |
| 3 | Age | 0.754 | -0.010 |
| 4 | Blood test: sodium | 0.753 | -0.011 |
| 5 | Blood test: chloride | 0.752 | -0.012 |
| 6 | Blood test: platelet count | 0.750 | -0.014 |
| 7 | Blood test: BUN | 0.749 | -0.015 |
| 8 | ICD-10 code R52 (pain) | 0.750 | -0.014 |
| 9 | Blood test: WBC count | 0.750 | -0.014 |
| 10 | Blood test: potassium | 0.749 | -0.015 |

ICD-10, International Classification Disease 10th revision; BUN, blood urea nitrogen; WBC, white blood cell.
