# Supplementary material for: Comparison of machine-learning and logistic regression models for prediction of 30-day unplanned readmission in electronic health records: A development and validation study
Source: PLOS Digit Health. 2024 Aug 20;3(8):e0000578. doi: 10.1371/journal.pdig.0000578 (PMC11335098; doi:10.1371/journal.pdig.0000578)
Supplement: Attachment — Submitted filename: Comments for PDIG-D-23-00402.pdf [file pdig.0000578.s012.pdf]

This study compares machine-learning models with logistic regression using electronic health records (EHRs) to predict unplanned readmissions across varying dataset complexities. Some questions need to be addressed properly.

1. The models labeled as "emerging," including gradient-boosted decision trees (GBDT), random forests (RF), and deep neural networks (DNN), are not accurately classified as newly emerging models in the context of machine learning.
2. The author should consider incorporating an updated review of relevant works from the past five years.
3. It is highly recommended to break down the "Results" section into distinct parts so that the readers can easily navigate and comprehend the findings, facilitating a clearer understanding of the study's outcomes and comparisons.
4. Provide more quantitative metrics or descriptive information to support conclusions. Are there any performance metrics other than C-statistic that would emphasize the advantages of GDBT over LR-LASSO?
5. While the ten most important variables in the GBDT model were identified, more detailed insights should be provided into the contribution of these variables to model performance and how they aid in understanding critical factors related to patient readmission risk.
6. What are the practical implications of the conclusions for actual clinical practice? How could these models be integrated into healthcare systems to improve patient care?
7. The resolution of the images in the article should be improved.
