# Supplementary material for: Comparison of machine-learning and logistic regression models for prediction of 30-day unplanned readmission in electronic health records: A development and validation study
Source: PLOS Digit Health. 2024 Aug 20;3(8):e0000578. doi: 10.1371/journal.pdig.0000578 (PMC11335098; doi:10.1371/journal.pdig.0000578)
Supplement: Attachment — Submitted filename: Response_to_Reviewers20240613re.docx [file pdig.0000578.s013.docx]

|  | **Editor comments** | **Response to the Editor** | **Place of change in the revised draft** |
| --- | --- | --- | --- |
| n/a | Please see the comments in the commented manuscripts and in the detailed sheets from the reviewers. I share their comments and hope for a good revision. | Thank you so much for reviewing our manuscript and offering valuable comments. We have responded to the reviewers’ comments in a point-by-point manner, as presented below. We believe that our revised draft is more suitable for publication in your journal. | n/a |
|  | **Comments by Reviewer #1** | **Response to Reviewer #1** |  |
| n/a | This study compares machine-learning models with logistic regression using electronic health records (EHRs) to predict unplanned readmissions across varying dataset complexities. Some questions need to be addressed properly. | We greatly appreciate your kind review and important suggestions. We have responded to your suggestions in a point-by-point manner, as presented below. | n/a |
| 1 | The models labeled as "emerging," including gradient-boosted decision trees (GBDT), random forests (RF), and deep neural networks (DNN), are not accurately classified as newly emerging models in the context of machine learning. | According to your suggestion, we have omitted “emerging” throughout the revised manuscript. | Throughout the draft |
| 2 | The author should consider incorporating an updated review of relevant works from the past five years. | We have conducted an updated review of relevant works and added the papers below in our revised manuscript:   - Talwar A, Lopez-Olivo MA, Huang Y, Ying L, Aparasu RR. Performance of advanced machine learning algorithms overlogistic regression in predicting hospital readmissions: A meta-analysis. Explor Res Clin Soc Pharm. 2023 Aug 10;11:100317. - Patel MS, Volpp KG, Small DS, Kanter GP, Park SH, Evans CN, Polsky D. Using remotely monitored patient activity patterns after hospital discharge to predict 30 day hospital readmission: a randomized trial. Sci Rep. 2023 May 22;13(1):8258. doi: 10.1038/s41598-023-35201-9.. - Wu T, Wei Y, Wu J, Yi B, Li H. Logistic regression technique is comparable to complex machine learning algorithms in predicting cognitive impairment related to post intensive care syndrome. Sci Rep. 2023 Feb 11;13(1):2485. doi: 10.1038/s41598-023-28421-6. - de Hond AAH, Kant IMJ, Honkoop PJ, Smith AD, Steyerberg EW, Sont JK. Machine learning did not beat logistic regression in time series prediction for severe asthma exacerbations. Sci Rep. 2022 Nov 27;12(1):20363. doi: 10.1038/s41598-022-24909-9. - Kang JS, Lee C, Song W, Choo W, Lee S, Lee S, et al. Risk prediction for malignant intraductal papillary mucinous neoplasm of the pancreas: logistic regression versus machine learning. Sci Rep. 2020 Nov 18;10(1):20140. doi: 10.1038/s41598-020-76974-7. | Page 16, line 16 – Page 17, line 1; page 17, lines 9-10; page 18, lines 16-17 |
| 3 | It is highly recommended to break down the "Results" section into distinct parts so that the readers can easily navigate and comprehend the findings, facilitating a clearer understanding of the study's outcomes and comparisons. | We have added the following subsections in the revised Results section: Characteristics of study participants, Characteristics of created datasets, Comparison of performances between prediction models, and Details of the prediction models in the data table with the largest number of variables. | Page 9, line 4; page 12, line 1; page 13, line 3; page 14 line 7 |
| 4 | Provide more quantitative metrics or descriptive information to support conclusions. Are there any performance metrics other than C-statistic that would emphasize the advantages of GDBT over LR-LASSO? | According to your suggestion, we have additionally estimated the indicators for overall fit (R2 Nagelkerke, R2 Cox-Snell, and Brier score) and calibration (calibration slope and calibration-in-the-large) to demonstrate the advantages of GDBT over LR-LASSO. | Page 13, line 17 – page 14 line 5; page 27, lines 7-9; S8 and S9 tables |
| 5 | While the ten most important variables in the GBDT model were identified, more detailed insights should be provided into the contribution of these variables to model performance and how they aid in understanding critical factors related to patient readmission risk. | In addition to the feature importance, we have shown the change in C-statistic by excluding the variables one by one to show the contribution of these variables to model performance. These variables changed the c-statistic, meaning that these variables are indeed good predictors for unplanned hospital readmission, and clinicians and clinical staff should pay attention to them. | Page 14, lines 16-18; page 27, lines 13-14; S11 table; page 18, lines 12-17 |
| 6 | What are the practical implications of the conclusions for actual clinical practice? How could these models be integrated into healthcare systems to improve patient care? | According to the results of the study, we believe that GBDT may be recommended for hospitals that apply clinical prediction models to their EHRs to identify patients at high risk of readmission. However, the benefit of GBDT over LR-LASSO was small, suggesting that “traditional regression models” such as LR models may also be acceptable, considering that they are more user-friendly and easier to interpret.  By integrating (one of) the models into healthcare systems, the patients at high risk for readmission may need to have their discharge postponed or receive intensive post-discharge follow-up as outpatients or remote monitoring with smartphones or wearable devices from home.  We have emphasized these points in our revised discussion section. | Page 18, lines 7-17 |
| 7 | The resolution of the images in the article should be improved. | We have replaced the images with high-resolution versions in our revised manuscript.  Once again, thank you very much for your kind review and important suggestions. | Figures 1-5 |
|  | **Comments by Reviewer #2** | **Response to Reviewer #2** |  |
| ① | Describe the source of data more in details. For instance, are the data maintained by the same system? What type of hospitals are they from? What sizes? What regions? | Thank you very much for your kind review and suggestions.  For the first question, the data are maintained by the same system at the Medical Data Vision (MDV) Co., and the data are collected in the same way as common data models as part of Diagnosis Procedure Combination (DPC) payment system in Japan.    Regarding the type of hospitals, the Japanese DPC payment systems are for acute care hospitals covered by the universal insurance system in Japan, and therefore, the study includes such acute care hospitals.  As we acknowledged in our limitation section in our first draft, the identities (including sizes and regions) of individual hospitals in the study are masked by MDV Co. to preserve privacy and data safety. However, we have cited a previous paper suggesting that the hospital volume (i.e., number of beds) of hospitals in MDV Co. tends to be larger than the average of hospitals in the Japanese DPC system.  We have clarified these points in our revised draft. | Page 21, lines 1-9 |
| 2 | "we intentionally created six pattens of datasets" – do you mean that you simulated data? i.e. generated artificial data? Or you sampled the original data in a certain way? Please elaborate and explain the method used for generating the datasets/ | By “We intentionally created six patterns of datasets having different numbers of binary variables (that ≥5% or ≥1% of patients, or ≥10 patients had) with and without blood-test results.”, we meant that we only changed the number of variables used for prediction, instead of generating artificial data or sampling patients.  To clarify this, first, we have added a sentence in the Method section:  “The six patterns of data tables only differ in the number of predictor variables; the included patients (i.e., 339,513 patients for derivation and 118,074 patients for validation) were the same in every data table.”  In addition, to avoid confusion, we have replaced “dataset(s)” with “data table(s)” throughout the draft. | Page 25, lines 7-9 |
| 3 | Calibration plots (Figure 4) – it would be more typical to arrange them with opposite axes – the predicted (in y axis) vs observed (in x axis) | It seems that the predicted (in the x-axis) vs observed (in the y-axis) is more common in medical journals. Indeed, the most recent educational paper in BMJ (Riley RD, et al. Evaluation of clinical prediction models (part 2): how to undertake an external validation study. BMJ. 2024 Jan 15;384:e074820. doi: 10.1136/bmj-2023-074820.) suggests showing the calibration figure in this order. Therefore, we prefer to keep our original Figure 4, unless the editor requests that we change it.  Once again, we appreciate your review and important suggestions. | n/a |
